# Leveraging Transfer Learning to Analyze Opinions, Attitudes, and Behavioral Intentions Toward COVID-19 Vaccines: Social Media Content and Temporal Analysis

**DOI:** 10.2196/30251

**Published:** 2021-08-10

**Authors:** Siru Liu, Jili Li, Jialin Liu

**Affiliations:** 1 Department of Biomedical Informatics Vanderbilt University Medical Center Nashville, TN United States; 2 West China Medical School Sichuan University Chengdu China; 3 Department of Medical Informatics West China Hospital Sichuan University Chengdu China

**Keywords:** vaccine, COVID-19, leveraging transfer learning, pandemic, infodemiology, infoveillance, public health, social media, content analysis, machine learning, online health

## Abstract

**Background:**

The COVID-19 vaccine is considered to be the most promising approach to alleviate the pandemic. However, in recent surveys, acceptance of the COVID-19 vaccine has been low. To design more effective outreach interventions, there is an urgent need to understand public perceptions of COVID-19 vaccines.

**Objective:**

Our objective was to analyze the potential of leveraging transfer learning to detect tweets containing opinions, attitudes, and behavioral intentions toward COVID-19 vaccines, and to explore temporal trends as well as automatically extract topics across a large number of tweets.

**Methods:**

We developed machine learning and transfer learning models to classify tweets, followed by temporal analysis and topic modeling on a dataset of COVID-19 vaccine–related tweets posted from November 1, 2020 to January 31, 2021. We used the F1 values as the primary outcome to compare the performance of machine learning and transfer learning models. The statistical values and *P* values from the Augmented Dickey-Fuller test were used to assess whether users’ perceptions changed over time. The main topics in tweets were extracted by latent Dirichlet allocation analysis.

**Results:**

We collected 2,678,372 tweets related to COVID-19 vaccines from 841,978 unique users and annotated 5000 tweets. The F1 values of transfer learning models were 0.792 (95% CI 0.789-0.795), 0.578 (95% CI 0.572-0.584), and 0.614 (95% CI 0.606-0.622) for these three tasks, which significantly outperformed the machine learning models (logistic regression, random forest, and support vector machine). The prevalence of tweets containing attitudes and behavioral intentions varied significantly over time. Specifically, tweets containing positive behavioral intentions increased significantly in December 2020. In addition, we selected tweets in the following categories: positive attitudes, negative attitudes, positive behavioral intentions, and negative behavioral intentions. We then identified 10 main topics and relevant terms for each category.

**Conclusions:**

Overall, we provided a method to automatically analyze the public understanding of COVID-19 vaccines from real-time data in social media, which can be used to tailor educational programs and other interventions to effectively promote the public acceptance of COVID-19 vaccines.

## Introduction

The outbreak of COVID-19 has affected 219 countries and territories with 102,083,344 confirmed cases causing 2,209,195 deaths as of January 31, 2021, as reported by the World Health Organization (WHO) [1]. As a significant global health threat, long-term control of COVID-19 relies on the development and acceptance of a preventive vaccine [2-4]. Fortunately, in November 2020, Pfizer-BioNTech and Moderna reported more than 95% efficacy of their vaccines [5], which were subsequently authorized by the US Food and Drug Administration (FDA) for emergency use. Since the preventive vaccine has been successfully developed, the current barrier is obtaining a sufficient proportion of the population to accept vaccines to slow the spread of the outbreak [6]. However, according to a recent survey, only 51% of 10,093 adults in the United States indicated that they would be willing to receive the COVID-19 vaccine when it becomes available [7], which would not achieve the recommended threshold of 70% to reach herd immunity [8].

Vaccine hesitancy, defined as “a behavior with delay in acceptance or refusal of vaccines despite available services,” was identified by the WHO as a global threat in 2019 [9]. The SAGA Working Group developed the Vaccine Hesitancy Determinant Matrix, including contextual influences (ie, related to historic, sociocultural, environmental, institutional, economic, or political factors), individual and group influences (ie, factors related to personal perception or social environment), and vaccine/vaccination-specific issues [10]. Unlike other common vaccines, the COVID-19 vaccines are associated with many factors that might amplify vaccine hesitancy [11,12]. Previous studies have reported widespread public concern about the rapid speed of vaccine development, novelty of the development technology (mRNA), unknown long-term side effects, and politicization of vaccines [13,14]. Furthermore, the social environment is polarized, with distrust of science among some groups and a plethora of conspiracies and misinformation about vaccines spreading across social media platforms [15,16]. For these reasons, it might be more difficult to achieve the coverage goal for COVID-19 vaccines. Therefore, it is urgent to efficiently collect information on public perceptions to tailor education materials for public and clinical guidance, which will enable primary care physicians to promote COVID-19 vaccines.

With the increased growth of internet-based applications, more people have begun sharing their opinions on social media platforms. In particular, during the current COVID-19 pandemic, people may increase their use of social media due to social distancing [11]. Social media is awash with virus conspiracies and misinformation [15]. Various social media platforms (eg, Facebook, Instagram, Reddit) are currently providing health information to researchers; among them, the Twitter platform has a more prominent role in gathering public perceptions on health care [17]. Twitter has become a good data source to collect real-time perceptions from a large-scale population for public health research. Over the past decades, researchers have used social media analytics tools to monitor public sentiment and communication patterns in a global pandemic crisis (eg, Ebola and Zika outbreaks) [18-20]. Mavragani [21] performed a time-series analysis on Google trends data and found a significant correlation between search interests with reported COVID-19 cases. Li et al [22] developed a taxonomy of Weibo posts on COVID-19 topics, and Liao et al [23] analyzed Weibo posts to identify public engagement and government responsiveness. Fadda et al [24] performed a content analysis to examine the extent of vaccine conspiracy theories reflected in tweets. Our study focused on the behavioral intentions related to COVID-19 vaccines, which is different from previous studies that performed a general analysis of COVID-19 tweets or vaccine conspiracy theories. Findings of this study could directly help researchers and policymakers to develop more targeted implementation strategies to improve acceptance rates of COVID-19 vaccines.

Machine learning and deep learning techniques have been used as efficient methods to detect public perceptions on social media platforms. In health care, researchers have developed deep learning models to perform longitudinal and geographic analyses to understand human papillomavirus (HPV) vaccine discussions [25]. These models also achieved good performance in predicting diagnosis or identifying patients in a high-risk group [26-28]. Transfer learning, as an emerging deep learning technique, has been applied to classify computed tomography images and notes. In transfer learning, a pretrained model is first used, which is then fine-tuned based on the specific datasets and tasks. Because the pretrained model already contains large-scale domain knowledge, the classification performance can achieve high values even with fine-tuning on relatively small datasets [29]. In this study, we applied Google’s bidirectional encoder representations from transformers (BERT) model as the pretrained model, which has achieved new state-of-the-art results in the natural language processing domain [29].

Although previous studies have explored additional knowledge in the context of other vaccines using machine learning and deep learning methods, several questions related to COVID-19 vaccines remain unknown: What is the prevalence of user opinions on a social media platform? How many tweets express positive/negative attitudes and behavioral intentions to take vaccines? Which topics are mostly associated with these contents? To answer these questions, we developed machine learning models (logistic regression, random forest, support vector machine) and transfer learning models to detect the content expressing user opinions, attitudes, and behavioral intentions toward COVID-19 vaccines. We then performed a temporal analysis to explore trends over time and developed probabilistic topic models to obtain the most important and valuable topics. We believe that this study will be of great benefit to the timely rollout of COVID-19 vaccines by extracting the latest public opinions, attitudes, and behavioral intentions that can help tailor promotion programs to fit different populations.

## Methods

### Study Overview

**Figure 1:**
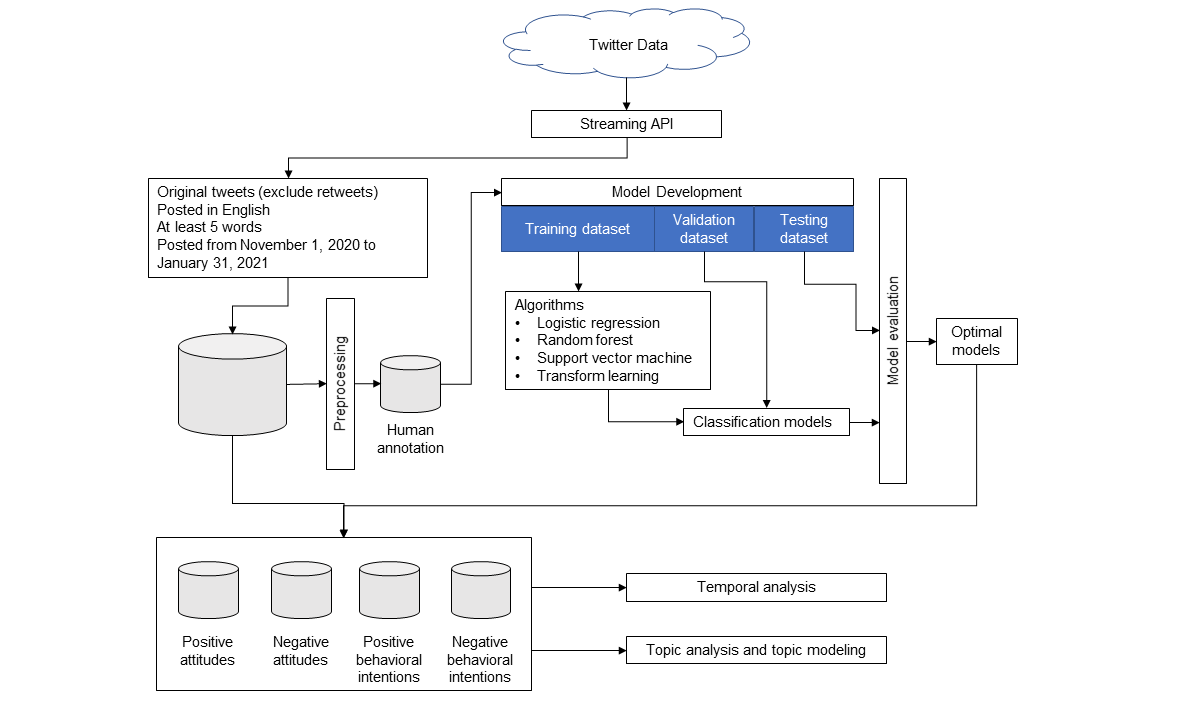
Overall study framework. API: application programming interface.

We collected tweets related to COVID-19 vaccines posted from November 1, 2020 to January 31, 2021, and annotated 5000 tweets as the gold standard. We developed machine learning and transfer learning models to classify tweets for three tasks: (1) opinions (yes, no); (2) attitudes (positive, negative, neutral); and (3) behavioral intentions (positive, negative, unknown). The above tasks all focused on COVID-19 vaccines. We then applied the models to predict unlabeled tweets and performed a temporal analysis to capture trends in the unlabeled tweets. In addition, we performed a topic analysis using word clouds and a latent Dirichlet allocation (LDA) model to further understand the content of tweets in the following categories: positive attitudes, negative attitudes, positive behavioral intentions, and negative behavioral intentions. The overall framework is shown in Figure 1.

### Data Collection

We used a combination of keywords and hashtags related to COVID-19 vaccines to collect tweets in English published from November 1, 2020 to January 31, 2021. We intentionally chose November, following the announcement of the first effective vaccine on November 9, 2020, to determine if the announcement of successful vaccine trial results might influence the perceptions of vaccines or vaccination. The search strategy employed the following search terms: “(#covid OR covid OR #covid19 OR covid19) AND (#vaccine OR vaccine OR #vacine OR vacine OR vaccinate OR immunization OR immune OR vax) since:2020-11-01 until:2021-01-31 lang:en.” We used snscrape and tweepy in Python 3 to collect data and to exclude retweets. To clean up the original tweets, we removed nonalphanumeric characters and converted the text to lowercase. We randomly selected 5000 tweets from November 1, 2020 to November 22, 2020, annotated by two independent reviewers (SL and JL) in batches of 200. Any annotation disagreements were discussed and adjudicated by the supervising investigators. For each tweet, we first labeled whether it included a user opinion toward the COVID-19 vaccines (yes or no). We considered a tweet to include an opinion about the COVID-19 vaccines if it met both of the following conditions: (1) targeted at the COVID-19 vaccines and (2) generated by a user. For the tweets that expressed user opinions toward the COVID-19 vaccines, we labeled the attitude (positive, negative, or neutral) and the behavioral intention (positive, negative, or unknown) toward COVID-19 vaccines. The attitude category used the traditional emotional polarity. The analysis of attitude was performed on the aspect level. If both positive and negative attitudes toward COVID-19 vaccines were present in the same tweet, we labeled it in the unknown category. The coding rules were iteratively developed by our group in which an independent review was performed, disagreements were discussed, and coding rules were revised. This process continued until the interrater agreement reached ≥0.80. The annotated corpus was used as a gold standard to train and evaluate the machine learning and transfer learning models.

### Model Development and Evaluation

For data preprocessing, we used the tweet-preprocessor package in Python 3 to remove URLs, hashtags, mentions, reserved words (eg, RT, FAV), emojis, smileys, and numbers in each tweet. We split the annotated dataset into three parts: training (60%), validation (20%), and testing (20%). The training and validation datasets were used to train models and select optimal hyperparameters through 5-fold cross-validation. We applied transfer learning using text frequency-inverse document frequency to compare traditional machine learning algorithms (logistic regression, random forest, and support vector machine) to transfer learning models. The machine learning models were developed using the scikit-learn package in Python 3.

For transfer learning, we used the BERT-base-cased as the pretrained language model and the “BERT for sequence classification” model as the pretrained classification model. Because the BERT model requires each sentence to be the same length, we padded each tweet with 64 tokens, as most tweets have lengths in this range. We then fine-tuned this model on the training and validation datasets using the Adam algorithm with weight decay (AdamW) as an optimizer. We performed three text classification tasks. We first developed a binary classifier to determine whether the tweets state an opinion related to the COVID-19 vaccines. We then developed two multiclass classifiers to categorize attitudes and behavioral intentions, respectively. The BERT models were generated using the huggingface package in Python 3. The models were developed with the Google Colab platform using a high-RAM GPU.

We evaluated the models on the testing dataset and report outcomes with 1000 rounds of bootstrapping. The primary outcome was the macro-F1 value and the secondary outcomes were recall, precision, and accuracy. We performed the Nemenyi test to compare the F1 values of traditional machine learning models and transfer learning models [30]. The model with the highest F1 value was considered the optimal model.

### Temporal Analysis

We applied the optimal models to predict the unlabeled data for 3 months starting from November 1, 2020. For the task of extracting opinions, we calculated the proportion of tweets classified as containing opinions to the total number of tweets posted each day about the COVID-19 vaccines. For the tasks of classifying attitudes and behavioral intentions toward the COVID-19 vaccines, we calculated the percentage of tweets predicted to exhibit a particular attitude or behavioral intention to all tweets indicating attitudes or behavioral intentions, respectively. To assess the statistical significance of variability over time, we performed the Augmented Dickey-Fuller (ADF) test [31] with a significance threshold of *P*<.05. The ADF test is a unit root test, which is commonly used to determine the stationarity of a time-series sample.

### Topic Analysis and Topic Modeling

To understand the content of tweets in each category, we used word clouds to illustrate the frequency of words appearing in the content. The more frequently used words have larger sizes, indicating more importance in the category [32]. Furthermore, we performed the LDA analysis to extract the main topics of discussion. LDA is a widely used unsupervised method that automatically clusters text based on content and identifies keywords in each topic through a probabilistic model [33,34]. We performed 5-fold cross-validation to tune hyperparameters in the LDA model (number of components and learning rate). After obtaining the results of the LDA models, we visualized extracted topics using the pyLDAvis library [35] in Python 3, which is an interactive visualization tool for displaying the distribution of topics and the top 30 most relevant terms with their weights in each topic.

## Results

### Performance of Classification Models

We annotated 5000 tweets from 4796 unique users with an average interrater reliability (κ) of 0.76. The prediction performances of models on the testing dataset using four different algorithms for three tasks are presented in Table 1. The transfer learning model significantly outperformed the machine learning models in identifying tweets that included opinions, attitudes, and behavioral intentions, achieving the highest F1 values of 0.792, 0.578, and 0.614, respectively.

**Table 1:** Metrics of transfer learning models and machine learning models in classifying tweets related to COVID-19 vaccines.

Task		Recall, mean (95% CI)	Precision, mean (95% CI)	F1, mean (95% CI)	Accuracy, mean (95% CI)
**Opinions**					
	BERT^a^	0.762 (0.759-0.766)	0.862 (0.858-0.866)	0.792^b^ (0.789-0.795)	0.854 (0.852-0.856)
	Logistic regression	0.774 (0.770-0.779)	0.757 (0.753-0.762)	0.764 (0.761-0.767)	0.807 (0.805-0.810)
	Random forest	0.754 (0.750-0.758)	0.732 (0.728-0.735)	0.740 (0.737-0.743)	0.783 (0.781-0.786)
	Support vector machine	0.767 (0.764-0.771)	0.752 (0.748-0.755)	0.758 (0.755-0.761)	0.803 (0.801-0.806)
**Attitudes**					
	BERT	0.529 (0.521-0.536)	0.698 (0.686-0.710)	0.578^b^ (0.572-0.584)	0.873 (0.871-0.875)
	Logistic regression	0.475 (0.468-0.482)	0.530 (0.520-0.541)	0.495 (0.490-0.500)	0.859 (0.856-0.861)
	Random forest	0.518 (0.511-0.526)	0.558 (0.545-0.570)	0.508 (0.502-0.514)	0.830 (0.827-0.833)
	Support vector machine	0.506 (0.498-0.514)	0.551 (0.541-0.562)	0.523 (0.517-0.530)	0.863 (0.860-0.865)
**Behavioral intentions**					
	BERT	0.562 (0.549-0.575)	0.734 (0.716-0.752)	0.614^b^ (0.606-0.622)	0.961 (0.960-0.962)
	Logistic regression	0.472 (0.461-0.483)	0.725 (0.699-0.752)	0.527 (0.519-0.536)	0.951 (0.949-0.952)
	Random forest	0.447 (0.437-0.457)	0.577 (0.543-0.611]	0.466 (0.457-0.476)	0.935 (0.934-0.937)
	Support vector machine	0.469 (0.458-0.479)	0.710 (0.684-0.737)	0.523 (0.513-0.533)	0.950 (0.948-0.951)

^a^BERT: Bidirectional encoder representations from transformers.

^b^*P*=.001 in the Nemenyi test.

### Temporal Analysis

**Figure 2:**
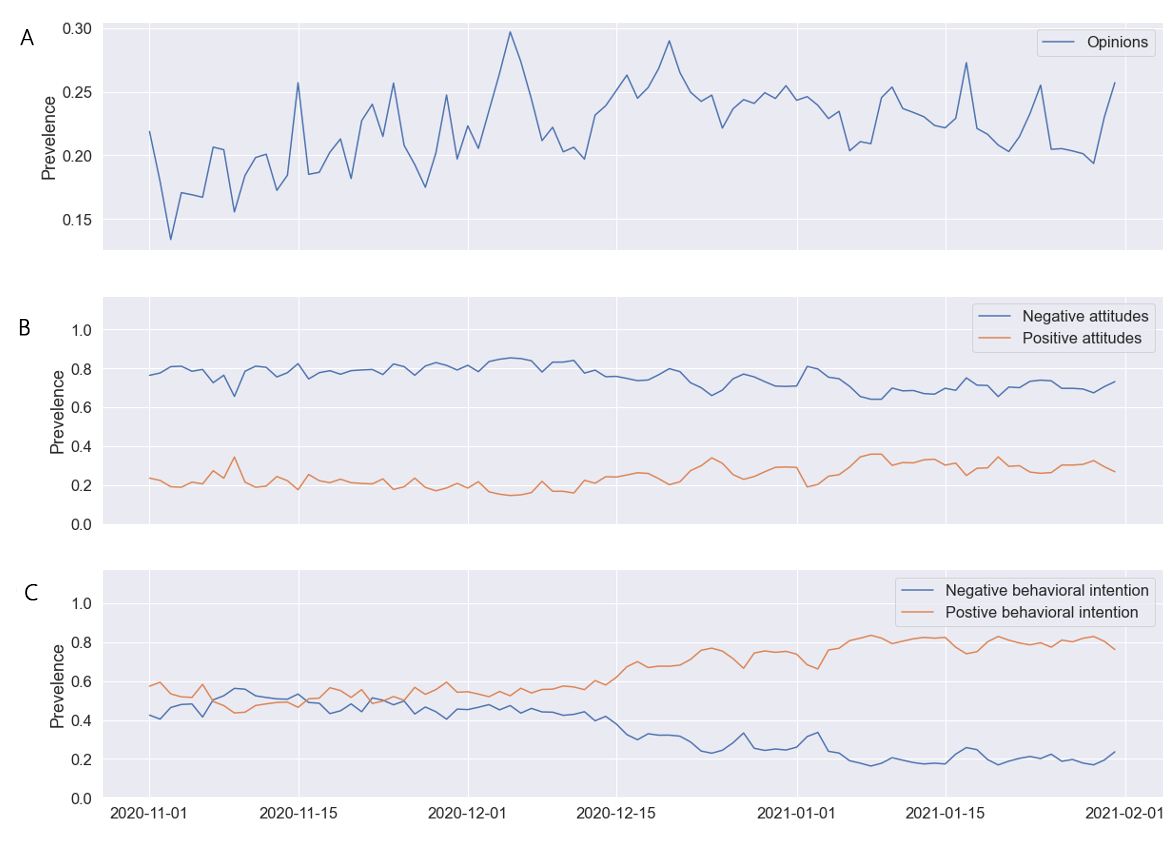
Distribution of the prevalence of the tweets containing opinions (A), attitudes (B), and behavioral intentions (C) about COVID-19 vaccines for each day from November 1, 2020 to January 31, 2021.

We collected 2,678,372 tweets related to COVID-19 vaccines posted by 841,978 unique users from November 1, 2020 to January 31, 2021. The daily prevalence distributions of opinions, attitudes, and behavioral intentions are shown in Figure 2. The daily prevalence of tweets expressing users’ opinions was 0.222 (95% CI 0.202-0.245). The ADF statistic was –4.341 (*P*<.001), indicating that the time-series data were stationary. This reflects that the prevalence of tweets expressing opinions did not change significantly over time. For tweets containing attitudes toward the COVID-19 vaccines, the rate of negative attitudes was 0.754 (95% CI 0.707-0.795), while the rate of positive attitudes was only 0.246 (95% CI 0.204-0.293). The daily prevalence of attitudes was nonstationary (ADF –1.137, *P*=.70), which indicated a significant change in users’ attitudes toward vaccines over time. Among tweets related to behavioral intentions, the rate of tweets indicating that users will not get vaccinated was 0.342 (95% CI 0.229-0.461), whereas the rate of tweets indicating that users will get vaccinated was 0.652 (95% CI 0.539-0.771). The behavioral intention prevalence was also nonstationary (ADF –0.980, *P*=.76), indicating that it varied significantly over time. Notably, we observed a substantial increase in the prevalence of tweets expressing positive behavioral intention starting from mid-December 2020.

### Topic Modeling and Analysis

#### Primary Domain Topics

**Figure 3:**
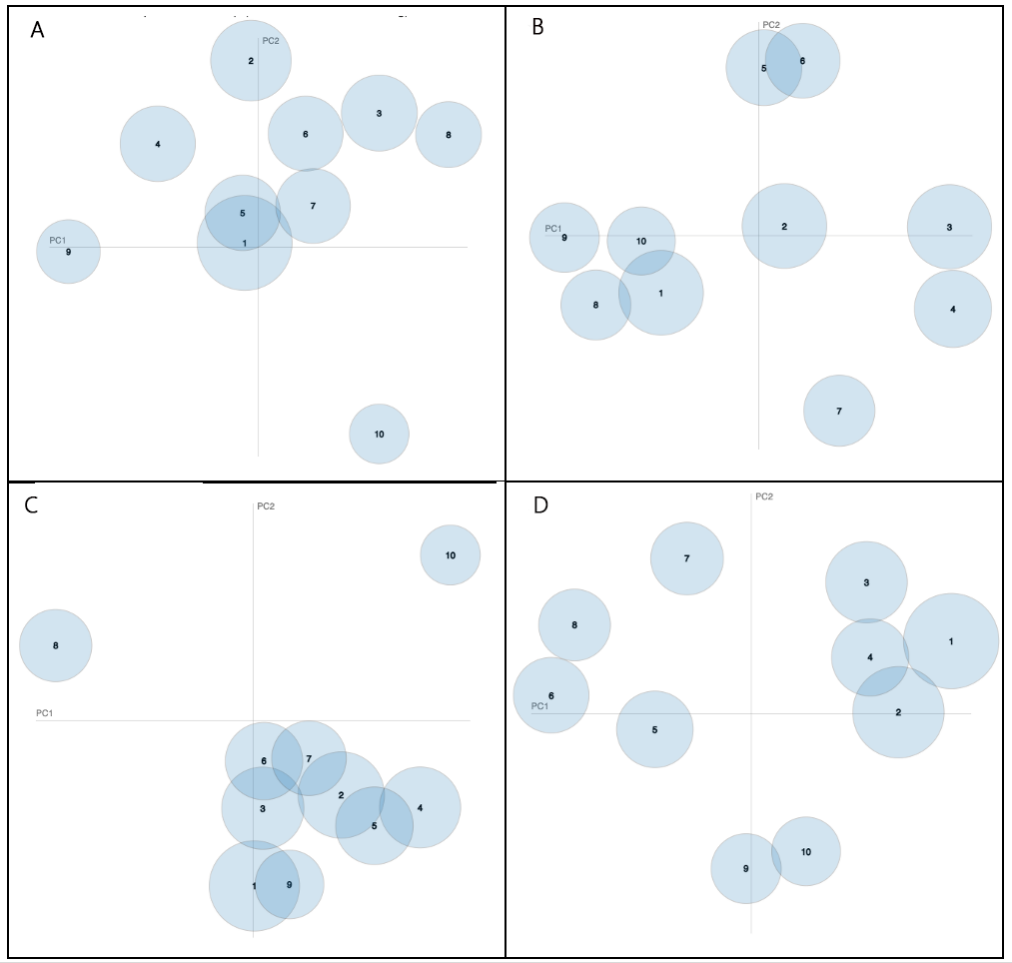
Intertopic distance maps for tweets that contained information in the following categories: negative attitudes (A), positive attitudes (B), negative behavioral intentions (C), and positive behavioral intentions (D).

After tuning hyperparameters of the LDA models, each model had 10 components (topics). Figure 3 presents intertopic distance maps generated by tweets containing positive/negative attitudes and positive/negative behavioral intentions. The size of bubbles represents the ratio of relevant tweets in that topic to the total number of tweets. In the following sections, we selected several domain topics for tweets in each category to describe the potential inferred themes based on identified relevant key terms. The overall top 15 keywords for each topic are listed in Textbox 1.

**Top 15 key terms for each topic.**

**Negative attitudes**

1. worry, prevent, covid, stop, need, spread, symptom, transmission, catch, people, reduce, infection, virus, eat, doesn

2. death, covid, case, people, rate, die, number, cause, population, test, trial, fear, report, survival, day

3. risk, covid, test, people, health, worker, trial, know, need, woman, work, child, pregnant, safe, age

4. effect, long, term, know, covid, bad, unknown, risk, affect, people, study, concern, damage, potential, impact

5. covid, year, make, anti, month, mask, rush, people, want, safe, need, just, know, sense, wear

6. covid, dose, use, virus, immune, antibody, body, immunity, trial, second, make, protein, cell, test, response

7. virus, new, covid, strain, effective, work, mutate, year, develop, mutation, research, cold, variant, different, make

8. covid, people, just, say, think, make, know, trust, want, cure, government, believe, thing, come

9. covid, die, people, life, chance, treatment, old, kill, effective, want, say, sick, save, safe, family

10. flu, covid, reaction, shot, drug, adverse, expect, people, shoot, allergic, just, high, bad, year, polio

**Positive attitudes**

1. covid, thank, work, great, today, day, make, worker, scientist, happy, mom, care, just, hard, nurse

2. covid, feel, effect, day, long, arm, just, little, work, fine, hour, term, good, excited, sore

3. safe, stay, end, covid, news, pandemic, effective, trial, good, amp, light, continue, home, hope, step

4. covid, hope, soon, look, forward, normal, life, hopefully, come, available, new, world, news, return, year

5. covid, good, year, just, time, wait, thing, hope, think, come, pray, love, wish, news, day

6. people, covid, want, need, know, die, risk, just, really, say, think, make, life, safe, fear

7. covid, dose, receive, today, grateful, second, family, feel, patient, able, thankful, protect, friend, happy, excited

8. flu, virus, covid, make, immune, fight, sure, body, new, immunity, just, strain, world, distribute, cause

9. mask, wear, covid, stop, social, spread, distancing, hand, catch, need, distance, people, virus, stay, help

10. covid, vaccinate, amp, case, symptom, prevent, ready, immunity, just, mean, virus, reduce, life, rate, infection

**Negative behavioral intentions**

1. covid, virus, stop, prevent, symptom, test, dose, immune, spread, mask, antibody, sick, just, catch, body

2. covid, flu, shot, shit, shoot, just, allow, work, win, scare, dead, year, virus, arm, sure

3. risk, covid, say, immune, make, high, virus, people, disease, just, healthy, sense, dangerous, case, good

4. want, covid, vaccinate, child, use, kill, kid, new, wait, way, cure, effective, doctor, just, people

5. covid, body, rate, vaccination, survival, choice, eat, mandatory, know, worry, life, fear, want, hear, need

6. covid, anti, just, tell, say, refuse, vaxxer, afraid, reason, people, stop, right, make, job, stupid

7. covid, year, trust, chance, inject, month, government, test, old, develop, cold, make, research, come

8. effect, know, long, term, covid, dna, affect, change, people, bad, rush, chance, unknown, study, test

9. people, covid, die, need, think, just, kill, family, care, damn, believe, say, real, death, chance

10. covid, force, try, reaction, people, bad, severe, look, allergic, medical, receive, say, fine, pay

**Positive behavioral intentions**

1. covid, people, want, just, think, say, know, mask, wear, make, really, ask, scare, right

2. covid, want, need, look, tomorrow, let, know, life, forward, ready, dose, morning, normal, receive, volunteer

3. covid, wait, long, turn, effect, line, term, finally, eat, excited, worried, afraid, use, drink, polio

4. just, dose, covid, second, got, day, effect, symptom, receive, fever, ache, hour, experience, headache, body

5. flu, covid, shot, year, time, bad, shoot, sick, immune, just, need, month, make, think, doctor

6. covid, arm, sore, sign, just, feel, today, hour, little, hurt, yesterday, injection, far, nervous, appointment

7. work, covid, home, thank, stay, patient, hospital, help, safe, care, protect, family, trial, receive, vaccinate

8. covid, risk, immune, die, people, virus, chance, high, know, need, vaccinate, healthy, live, just, catch

9. covid, today, hope, mom, test, dose, happy, able, soon, dad, positive, receive, good, grateful

10. feel, covid, day, week, fine, great, make, shit, ago, better, worker, body, job, good, healthcare

#### Attitudes

**Figure 4:**
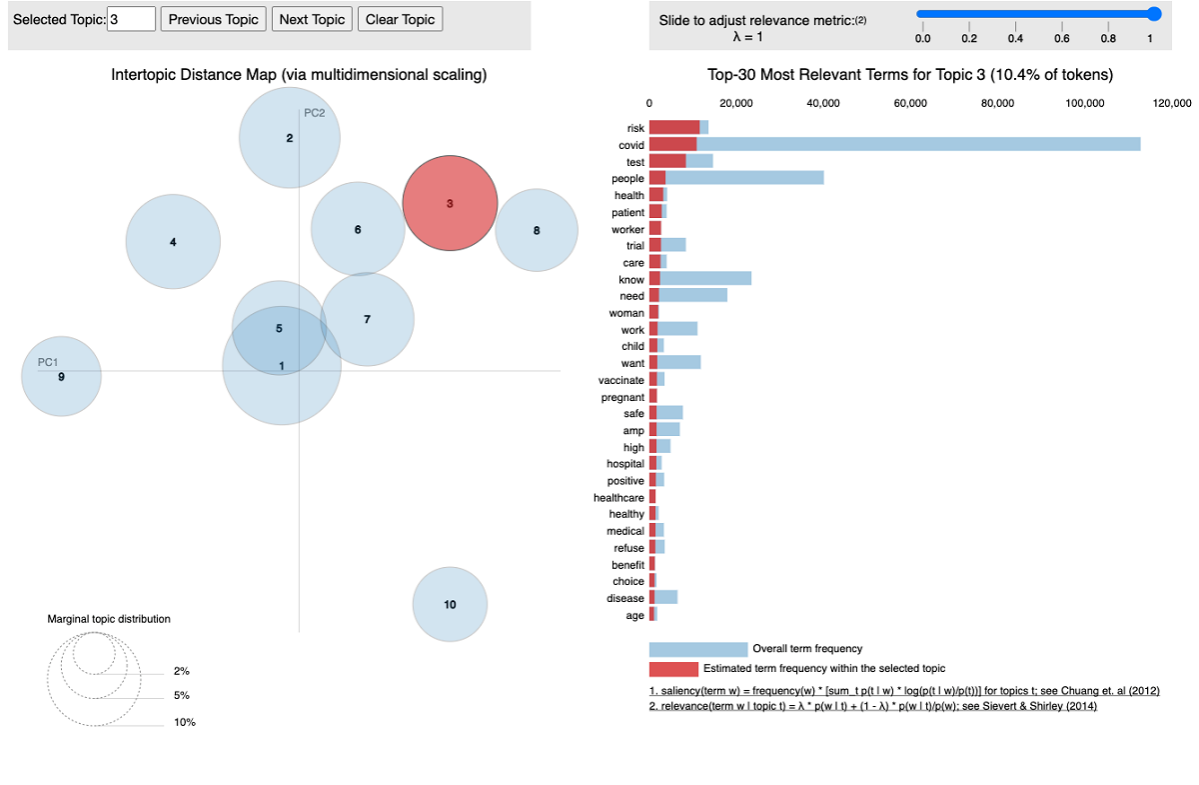
PyLDAvis visualization highlighting the top 30 relevant keywords for a topic found in the tweets that contained negative attitudes toward COVID-19 vaccines.

Ten topics were extracted among the tweets that contained negative attitudes. The interactive display interface of pyLDAvis is shown in Figure 4. The left panel shows the distribution of topics, and we could choose the topic we wanted to analyze by clicking on the bubble (eg, topic 3 highlighted in Figure 4), while the right panel lists the top 30 relevant terms and their weights contributing to the selected topic. Some important keywords contained in topic 3 were “risk,” “test,” “child,” “safe,” “pregnant,” “disease,” and “age.” Topic 3 summarized that users with negative attitudes were concerned about the safety issues of the COVID-19 vaccines, especially about the risks for certain populations such as children, pregnant women, and patients with immune diseases. Other topics reflected concerns about unknown side effects (topic 4) and rushing the development process (topic 5). Some users even questioned the existence of COVID-19 or COVID-19 vaccines and indicated a lack of trust in the government or scientists (topic 8). In addition, some users feared that the virus mutation would render the vaccine ineffective (topic 7) and thus had negative attitudes toward vaccines.

For tweets containing positive attitudes, in a dominant topic (topic 3), relevant key terms included “safe,” “stay,” “end,” pandemic,” “news,” “effective,” “trial,” “continue,” and “hope.” This indicates that some positive attitudes might be derived from news of effective trial results and some users hoped that COVID-19 vaccines could end the pandemic. Relevant terms for topic 4 were “hope,” “normal,” “life,” “return,” “start,” “new,” “world,” and “great.” Tweets in topic 4 showed that some users expressed positive attitudes toward vaccines because of the desire to return to a normal life.

#### Behavioral Intentions

For tweets containing negative behavioral intentions, topics 8 and 10 clustered independently; however, other topics showed some degree of mutual inclusiveness, indicating that similarities existed in those topics. Key terms for topic 8 were “effect,” “know,” “long,” “term,” “DNA,” “unknown,” and “rush.” This topic reflected that some users’ negative behavioral intentions came from the concerns of the long-term and unknown side effects of COVID-19 vaccines. As another unique topic, the most relevant terms for topic 10 were “force,” “reaction,” “bad,” “allergic,” “pay,” “adverse,” and “government.” This analysis highlighted that some users mentioned that they would not take the vaccine if it was forced on them by the government. Others worried about the adverse reactions to the COVID-19 vaccines. Some users compared COVID-19 to influenza and mentioned that because they had not previously been vaccinated against influenza, there was also no need to vaccinate against a disease they mistakenly thought had the same low lethality (topic 2). Other users reported that their immune system could naturally help them fight the virus.

For tweets containing positive behavioral intentions, mutual inclusivity existed among topics 1-4 and between topics 9 and 10. Other topics clustered independently. In topic 8, the keywords were “risk,” “immune,” “healthy,” “antibody,” and “immunity.” In this topic, users would like to become immune to the virus causing COVID-19 and stay healthy by being vaccinated.

## Discussion

### Principal Findings

In this study, we provided an annotated dataset with 5000 COVID-19 vaccine–related tweets with labels supporting three classification tasks (opinions, attitudes, and behavioral intentions). We assessed that transfer learning could be used to analyze COVID-19 vaccine content tweets and proved that they outperformed common machine learning models. We analyzed the temporal trends and topics in the COVID-19 vaccine–related tweets posted over a 3-month period (from November 1, 2020 to January 31, 2021). The prevalence of tweets containing positive behavioral intentions increased over time. The word clouds and the LDA analysis proved to be efficient tools to understand topics for tweets in each category.

Transfer learning is now widely used to analyze social media content. Some researchers have applied transfer learning with datasets of tweets related to COVID-19 [36-38] rather than focusing on tweets related to the vaccines developed for this disease. Researchers have analyzed tweets related to other vaccines such as HPV vaccines [25]. However, few studies have annotated tweets containing content about COVID-19 vaccines or developed models to understand public perceptions on COVID-19 vaccines from social media. For example, Levy et al [36] applied cross-lingual transfer learning to model COVID-19 outbreak patterns in one country, and then utilized the model to predict the spread of the disease in another country with a strong Spearman correlation (0.850). A classification model based on transfer learning developed by Spangher et al [37] was able to categorize policy announcements of COVID-19 using event extraction, with an F1 score of 0.770. To identify informative tweets related to COVID-19, Tasneem et al [38] proposed a unified architecture to combine transfer learning with hand-crafted features, achieving an F1 score of 0.820. Du et al [25] used deep learning models to categorize HPV vaccine–related tweets with constructs in the health belief model and theory of planned behavior models, and obtained F1 scores ranging from 0.681 to 0.942. Our study is the first to apply transfer learning models to analyze the public’s attitudes and behavioral intentions toward COVID-19 vaccines. Our model also achieved good performance, with F1 scores ranging from 0.579 to 0.792. In addition, we provided an annotated dataset with 5000 tweets, each labeled according to whether the tweet contained users’ opinions, attitudes, or behavioral intentions on COVID-19 vaccines. This dataset can be used for further research on social media content related to the COVID-19 vaccines.

Several researchers have applied the Valence Aware Dictionary and Sentiment Reasoner (VADER) tool [39,40], machine learning [41], and deep learning [42] to perform sentiment analysis on COVID-19–related tweets. Chandrasekaran et al [39] and Yin et al [40] employed the VADER tool to calculate the polarity of sentiment in COVID-19–related tweets posted in the first half of 2020. Both of these studies reported that the proportion of positive tweets was higher than that of negative tweets in general. However, Chandrasekaran et al [39] determined that negative tweets were dominant in the themes of symptoms and spread in cases. Li et al [42] used deep learning to identify fear and sadness emotions mentioned in COVID-19–related tweets to analyze the public’s mental health status, and reported area under the receiver operating characteristic curve values ranging from 0.681 to 0.739. Chakraborty et al [41] developed machine learning models with Gaussian membership function–based fuzzy rules to classify sentiment in COVID-19–related tweets, obtaining accuracy values ranging from 0.526 to 0.814. Although these previous studies have classified sentiment in COVID-19–related tweets, our study differs with respect to the task of classifying attitudes toward COVID-19 vaccines. We not only focused on the sentiment of tweets but also simultaneously examined whether the object of the sentiment was the COVID-19 vaccine. During annotation, we noticed that some tweets contained positive words used to describe what would happen after the vaccine rollout but also stated negative attitudes toward the vaccine itself, such as lack of trust and rushing.

Temporal analysis and topic modeling provide an efficient approach to monitor public perceptions of the COVID-19 vaccines on social media platforms. The following events could explain the significant increase in the prevalence of positive behavioral intentions in mid-December. For example, the FDA issued Pfizer-BioNTech COVID-19 vaccines on December 11, 2020, turning the vaccines from a hypothetical situation into a reality. The United States launched its rollout to high-risk health care facilities on December 14, 2020. A large number of health care workers and influential figures such as Joe Biden received COVID-19 vaccines to increase public confidence. This also suggests that more people might be willing to be vaccinated after successful vaccine development and a large-scale rollout. Indeed, social influence has been shown to positively affect the acceptance rate [43]. At the same time, this increase in positive behavioral intentions could also generate a positive social influence, which could lead to a higher vaccine acceptance rate. Therefore, the low acceptance rate of COVID-19 vaccines reported in the surveys conducted prior to December 2020 might not accurately reflect the current situation. Researchers should consider resurveying the public’s intention to receive the vaccination. Key terms identified in topic modeling could provide the needed guidance to design or optimize vaccine promotion interventions (eg, education materials). COVID-19 vaccine promotion strategies need to solve concerns on side effects and long-term safety issues, virus mutation, and the difference between COVID-19 and the flu. Moreover, promotion strategies should highlight the chance to return to normal life and stay healthy after being vaccinated for COVID-19.

### Limitations

This study has several limitations. First, users of the Twitter platform are not representative of the entire public. The Twitter platform is usually considered to gather more antivaccinators and spread misinformation. This group of users is the main subgroup of the population with sentiments of vaccine hesitancy and should therefore be one of the main targets to receive vaccine education. Compared to other populations, they tend to question vaccines from specific perspectives such as the presence of microchips in vaccines [44] and the use of human embryos in the process of developing vaccines. Understanding their perceptions is a necessary step to tailor vaccine promotion education materials, which would provide a better chance of effectively changing their behavior. Second, some topics extracted from the topic modeling might be difficult to infer accurately using relevant terms. In addition, given the complex situation of behavioral intentions toward COVID-19 vaccines mentioned in the tweets, further qualitative studies (eg, content analysis) combined with theoretical models are needed to understand why some people will not get the vaccines from psychological aspects. Third, we applied the “BERT-base-uncase” as the language model. Recently, researchers have developed a transformer-based model COVID-Twitter-BERT (CT-BERT) model, which was pretrained on COVID-19–related tweets [45], and they also expected to obtain performance gains when applying the CT-BERT model on health care content tweets. The impact of using the CT-BERT model on our classification tasks is unknown. Fourth, the annotated corpus included 5000 tweets. If more annotated data could be collected, the performance of the model might be improved.

For future work, we will perform a theory-based content analysis to gain insight into the reasons that led to the changes in behavioral intentions we noted in the temporal analysis. Using the transfer learning model in this study, researchers can automatically collect tweets containing COVID-19 vaccine–related behavioral intentions and systematically analyze the data through a theoretical model (eg, Capability, Opportunity, Motivation, Behavior model [12,46]) to promote timely promotion strategies. In addition, researchers can extract individual characteristics from the user profile and perform statistical analysis to determine the relationship between individual characteristics and their behavioral intention toward COVID-19 vaccines.

### Conclusion

In this study, we presented an annotated corpus of 5000 tweets and analyzed the potential to use transfer learning with a pretrained BERT model to automatically identify public opinions, behavioral intentions, and attitudes toward COVID-19 vaccines from social media. We demonstrated that transfer learning models outperformed traditional machine learning models in general. In addition, we explored the temporal trends of the public’s change in attitudes and behavioral intentions on a larger dataset with 2,678,372 tweets from November 1, 2020 to January 31, 2021. We found that the LDA technique is useful to extract topics from identified tweets. Overall, we provided an automatic method to analyze the public’s understanding of COVID-19 vaccines from real-time data, which could be used to tailor education programs and other interventions to promote COVID-19 vaccine acceptance urgently.

## Abbreviations

ADF: Augmented Dickey-Fuller
BERT: bidirectional encoder representations from transformers
CT-BERT: COVID-Twitter-bidirectional encoder representations from transformers
FDA: Food and Drug Association
HPV: human papillomavirus vaccine
LDA: latent Dirichlet allocation
VADER: Valence Aware Dictionary and Sentiment Reasoner
WHO: World Health Organization
